# Real-Time Low-Cost Traffic Monitoring Based on Quantized Convolutional Neural Networks for the CNOSSOS-EU Noise Model

**DOI:** 10.3390/s26051736

**Published:** 2026-03-09

**Authors:** Domenico Profumo, Gonzalo de León, Alessandro Monticelli, Luca Fredianelli, Gaetano Licitra

**Affiliations:** 1 IPOOL S.R.L., Via Antonio Cocchi, 3, 56121 Pisa, Italy; domenico.profumo@i-pool.it; 2 Earth Sciences Department, University of Pisa, Via Santa Maria, 53, 56126 Pisa, Italy; gondleon@gmail.com; 3 Department of Physics “E. Fermi”, University of Pisa, Largo Bruno Pontecorvo, 3, 56127 Pisa, Italy; alessandromonticelli.1@gmail.com; 4 Institute for Chemical-Physical Processes of the Italian Research Council (CNR-IPCF), Via Moruzzi, 1, 56100 Pisa, Italy; 5 Pisa Department, Environmental Protection Agency of Tuscany (ARPAT), Via Vittorio Veneto, 27, 56127 Pisa, Italy; g.licitra@arpat.toscana.it

**Keywords:** real-time vehicle detection, quantized convolutional neural networks, CNOSSOS-EU classification, edge computing, traffic flow monitoring, noise assessment support

## Abstract

Accurate urban noise mapping requires granular traffic flow characterization aligned with specific acoustic models, such as CNOSSOS-EU. Existing monitoring solutions often lack the specific categorization capabilities, cost-effectiveness, or flexibility required for large-scale deployment in resource-constrained environments. To address this challenge, the present study describes the development of a real-time multi-vehicle recognition system based on low-cost edge computing hardware, specifically a Raspberry Pi 4 coupled with a Coral TPU accelerator. The proposed methodology integrates a quantized YOLOv8 convolutional neural network (CNN) with a tracking algorithm to enable real-time detection and classification of vehicles into five distinct classes, allowing for precise aggregation according to CNOSSOS-EU standards. The model was trained on a proprietary dataset of 15,000 images and subjected to 8-bit post-training quantization to optimize inference speed. Experimental results demonstrate that the system achieves an inference speed of 14 FPS and a mean Average Precision (mAP@50) of 92.2% in daytime conditions, maintaining robust performance on embedded devices. In a real-world case study, the proposed system significantly outperformed a commercial traffic monitoring solution, achieving a weighted percentage error of just 6.6% compared to the commercial system’s 59.9%, effectively bridging the gap between manual counting accuracy (1.4% error) and automated efficiency.

## 1. Introduction

In order to correctly address some of the most important environmental challenges in urban areas, it is necessary to thoroughly characterize the composition and volume of traffic flows [1,2,3]. The utility of such a task is twofold: on one hand, it provides valuable insight into the driving conditions of different areas; on the other hand, it is essential for evaluating the acoustic climate according to standardized models, such as the Common Noise Assessment Methods in Europe (CNOSSOS-EU) [4].

Unlike general traffic monitoring, acoustic modeling requires a high level of granularity, as noise emissions vary significantly between different vehicle classes (e.g., light vehicles vs. heavy trucks or motorcycles). Therefore, the ability to accurately distinguish between vehicle categories is not merely a technical detail but a fundamental requirement for the accuracy of urban noise maps. However, traditional methods such as inductive loops or pneumatic tubes often lack the capability to classify vehicles with the required precision [5], while high-end computer vision systems are typically too expensive and power-demanding for capillary, large-scale deployment.

The development of intelligent systems for traffic data acquisition has been driven by recent innovations [6,7,8]. Moreover, connected vehicle scenarios have also motivated research on resilient traffic control strategies under cyber-physical threats, including self-stabilizing approaches integrated into continuum traffic models [9]. Deep learning approaches have proven effective not only for road traffic but have also been successfully adapted for safety and monitoring in other transport domains, such as foreign object detection in urban rail transit systems [10]. These systems, which are often designed for general traffic management, present limitations when applied to acoustic modeling, as they may be suboptimal for this specific purpose or too expensive for large-scale implementation. A significant step forward in this field was previously reported in [11], where a low-cost traffic data collection system was developed. That system employed an innovative approach integrating low-cost sensors with video recognition algorithms to classify vehicles according to CNOSSOS-EU guidelines. More recently, this methodology has been further refined with the EAgLE 3.0 model [12], which improved the validation of smart road traffic noise modeling by enhancing vehicle tracking capabilities through advanced image recognition techniques. However, despite its classification accuracy, the previous iteration relied on offline processing and lacked the computational efficiency required for autonomous, real-time edge operation. This limitation highlighted the necessity to enhance the robustness of the instrumentation under variable environmental conditions and to optimize the algorithms for real-time data processing directly on the device.

The primary objective of this research is to bridge this gap by developing a real-time, energy-efficient, and low-cost multi-vehicle recognition system capable of running entirely on embedded hardware. By leveraging a quantized Convolutional Neural Network (CNN) and a lightweight tracking algorithm, the proposed system ensures precise categorization compatible with acoustic noise modeling requirements without the need for external processing units.

Consequently, the main contributions of this work are presented as follows. First, the study details the development and optimization of a quantized object detection model (specifically YOLOv8n), implemented via post-training quantization to enable real-time inference on a Raspberry Pi 4 Model B (Raspberry Pi Ltd., Cambridge, UK) paired with a Coral USB Accelerator (Google LLC, Mountain View, CA, USA). Second, the network was custom-trained to distinguish five specific vehicle classes: light vehicles, medium-heavy vehicles, heavy vehicles, buses, and motorcycles. This high-resolution classification allows for flexible mapping to the standard CNOSSOS-EU categories, specifically by aggregating the separate ‘bus’ and ‘heavy’ detections into the acoustic Category 3 (heavy vehicles with more than two axles), thus ensuring high fidelity in noise modeling. Third, the system integrates a computationally efficient tracking algorithm to perform accurate vehicle counting and flow analysis directly on the edge device. Finally, the reliability of the proposed solution is validated through a comparative analysis against a commercial traffic monitoring system and ground-truth manual counts, demonstrating superior accuracy in complex urban environments.

These findings constitute a significant advancement in facilitating more efficient and accessible traffic measurement, thereby establishing the foundation for increasingly precise and scalable acoustic modeling. The proposed systems are characterized by their compact nature, ease of replication, cost-effectiveness, and the potential to foster a more sustainable and informed management of traffic noise issues.

The paper is structured as follows: Section 2 describes the theoretical background and characterizing features of the models used for object detection. Section 3 details the development of the detection system, including the experimental setup, dataset creation, and the quantization process. In Section 4, the experimental results are presented and discussed, including a real-world case study. Finally, Section 5 concludes the study by summarizing the results and outlining possible future developments.

## 2. Theoretical Background

Automated traffic monitoring relies on the integration of object detection and multi-object tracking (MOT) to accurately identify and track vehicles across sequential frames in video streams. Object detection localizes and classifies vehicles in each frame, but it does not establish temporal associations between detections, which are essential for movement analysis, vehicle counting, and trajectory estimation. To address this, MOT algorithms associate detected objects across frames, maintaining unique identities for each vehicle over time. The effectiveness of a traffic monitoring system depends on the accuracy and efficiency of both detection and tracking components, as challenges such as occlusions, motion blur, and overlapping objects can significantly impact performance.

In real-time edge computing environments, balancing precision and computational efficiency is crucial. High-performance object detectors provide fast and accurate detections, while tracking algorithms offer different trade-offs between tracking accuracy and computational complexity. Selecting an optimal combination is key to ensuring real-time performance and robust tracking in dynamic traffic conditions.

### 2.1. Object Detectors

Vehicle detection plays a critical role in traffic monitoring, enabling applications such as traffic flow assessment [13] and urban noise estimation [14]. While traditional methods like the Gaussian Mixture Model (GMM) have shown promise, they struggle with maintaining robust accuracy across varying illumination and weather conditions [15]. Recent advances in deep learning-based object detection have led to more reliable and scalable solutions, significantly improving detection accuracy and generalization in diverse environments. Object detection architectures are generally categorized into two-stage detectors, which offer high precision but are computationally demanding, and single-shot detectors (SSDs), which prioritize inference speed by performing detection and classification in a single step [16].

This study focuses on single-shot detectors, which are more suitable for real-time and embedded applications due to their lower computational overhead. Notable single-shot detection architectures include SSD [17], EfficientDet [18], CenterNet [19], RetinaNet [20], and YOLO (You Only Look Once) [21]. Among these, YOLOv8, SSD MobileNet v2, and SSD/FPN-based models were selected to be analyzed based on detection accuracy, processing speed, and hardware compatibility. Given the system’s constraints—a Raspberry Pi 4 microprocessor coupled with a Coral TPU accelerator—these models offer the best balance between real-time inference and computational efficiency. It is worth noting that while this study focuses on established detectors, the field is rapidly advancing towards hybrid architectures combining CNNs and Transformers to enhance fine-grained recognition capabilities, as demonstrated in recent biometric studies [22], which pave the way for future improvements in complex vehicle classification tasks.

#### 2.1.1. SSD Mobilenet v2

Single Shot Detector (SSD) networks are efficient object detection models that simultaneously classify and localize objects within an image. They typically use a VGG-16-based backbone for feature extraction, followed by additional convolutional layers to process deeper image features [23]. The detection pipeline includes a prediction layer that outputs class probabilities and bounding box coordinates, with non-maximum suppression (NMS) applied to filter redundant detections. While SSDs are well-suited for real-time applications, their computational demands pose challenges for deployment on embedded systems.

To improve efficiency, SSD architectures can be optimized with MobileNet-based encodings, replacing standard convolutions with depthwise separable convolutions. This reduces computational complexity without reducing detection accuracy [24]. MobileNet v2 has shown comparable mAP scores to its predecessor while significantly reducing model size, making it an ideal candidate for edge-based applications [25].

Further enhancements can be achieved using Feature Pyramid Networks (FPNs), which refine object detection by integrating multi-scale features from both bottom-up and top-down pathways [26]. Originally designed for Region Proposal Networks (RPNs), FPNs have since been incorporated into SSD/MobileNet architectures. Preliminary tests on a Coral-accelerated Raspberry Pi 4 demonstrated increased detection accuracy, albeit at the cost of reduced classification speed due to added computational overhead.

#### 2.1.2. YOLOv8

The YOLO (You Only Look Once) framework, introduced by [21], revolutionized object detection by enabling simultaneous localization and classification in a single forward pass. Unlike traditional two-stage detectors and sliding window-based classifiers, which require multiple forward passes and region proposals, YOLO streamlined the detection pipeline, significantly improving computational efficiency. By leveraging pointwise convolutions, YOLO reduces the number of channels in feature maps, minimizing model complexity without sacrificing accuracy. Over the years, various iterations of YOLO have been developed, with this study specifically focusing on YOLOv8 by Ultralytics, the latest and most optimized version available at the time of testing.

YOLOv8 introduces C2f convolutional structures, which enhance feature extraction by fusing high-level semantic information with spatial details. Additionally, it incorporates distributed prediction heads [27], where each branch is optimized for a specific task, improving detection accuracy. Bounding box regression in YOLOv8 is refined using Complete Intersection over Union (CIoU) loss [28], while Distributed Focal Loss (DFL) [29] helps improve object localization. Classification accuracy is ensured using a binary cross-entropy loss function, enhancing the model’s robustness in real-world applications.

To accommodate varying computational constraints, YOLOv8 is available in five different sizes: YOLOv8n (nano), YOLOv8s (small), YOLOv8m (medium), YOLOv8l (large), and YOLOv8x (extra-large). The YOLOv8n variant is specifically optimized for minimal computational overhead, making it ideal for embedded systems and mobile applications. Given the need for real-time inference on resource-limited hardware, this study focuses on YOLOv8n, which offers the best balance between efficiency and accuracy, making it particularly suitable for edge computing in traffic monitoring systems. Since the adoption of YOLOv8 for this study, the Ultralytics YOLO model zoo [30] has continued to evolve rapidly, introducing subsequent architectures aimed at improving accuracy, training strategies, and deployment characteristics. Notable examples include YOLOv9, YOLOv10, and YOLO11, as well as the recently released YOLO26 [31], which adopts an end-to-end, NMS-free design and streamlines model export and deployment on embedded accelerators. While these developments are promising, YOLOv8n remains a suitable choice for the present work because it provides a well-established trade-off between accuracy and latency and relies on a mature deployment toolchain for embedded inference with post-training optimization. This aspect is particularly relevant for continuous traffic monitoring, where stable real-time performance and reproducible on-edge deployment are primary requirements.

#### 2.1.3. EfficientDet

EfficientDet [18] is an object detection architecture that leverages EfficientNet [32] as its backbone, providing a balance between high detection accuracy and computational efficiency. EfficientNet itself is an image classification model designed to maximize accuracy while maintaining a lightweight structure, achieved through automated architecture optimization. Its development was guided by Neural Architecture Search (NAS), which determined the optimal depth, width, and resolution parameters for improved performance.

For object detection, EfficientDet extracts feature maps from layers 3 to 7 of its EfficientNet backbone. These feature maps are then merged and processed using a Bidirectional Feature Pyramid Network (BiFPN), an enhanced version of the traditional Feature Pyramid Network (FPN).

BiFPNs introduce a bidirectional feature fusion mechanism, enabling cross-scale feature integration. Unlike conventional FPNs, which only allow top-down feature fusion, BiFPNs enhance feature propagation by incorporating both top-down and bottom-up pathways, ensuring better multi-scale feature representation. Additionally, BiFPNs implement weighted feature fusion, dynamically assigning importance to different feature maps based on their relevance to the detection task.

Once processed through the BiFPN, the extracted features are passed to the classification and regression heads, which predict the bounding box coordinates and class labels for each detected object. Through its lightweight yet highly efficient design, EfficientDet has proven to be an effective solution for real-time object detection, particularly in resource-constrained environments.

#### 2.1.4. RetinaNet

RetinaNet ([33]) was introduced as an object detection architecture alongside a novel loss function designed to mitigate class imbalance issues. The architecture consists of three main components: (1) a backbone network, typically a ResNet-based model (e.g., ResNet-50 or ResNet-101), responsible for feature extraction; (2) a Feature Pyramid Network (FPN) for multi-scale feature fusion; and (3) classification and regression heads, which predict object classes and bounding box coordinates.

One of the key challenges in object detection is class imbalance, where dominant classes overshadow less frequent ones, leading to suboptimal training performance. RetinaNet addresses this issue through the introduction of Focal Loss, an advanced loss function that adjusts the contribution of easy and hard examples during training through an exponential parameter. Focal Loss is defined as:

(1) $$FL\left(p_{t}\right)=-{\left(1-p_{t}\right)}^{\gamma }\log(p_{t})$$

where $p_{t}$ is the prediction probability associated with the standard cross entropy loss. A modulating factor, dependent on a parameter γ is thus introduced to address the effects associated with class imbalance.

#### 2.1.5. CenterNet

CenterNet is an object detection architecture that performs bounding box regression and classification without relying on anchor boxes or non-maximum suppression (NMS) post-processing. Traditional object detection methods often utilize anchor boxes, which require the model to generate multiple candidate bounding boxes per object. These outputs are then compared using the Intersection over Union (IoU) metric, where overlapping boxes with high IoU values are grouped together as part of the same detection. Among these candidates, only the bounding box with the highest confidence score is retained, while the others are suppressed through NMS, reducing redundancy.

To mitigate the computational overhead associated with anchor-based architectures, CenterNet adopts an alternative approach [34] based on keypoints and heatmaps. Instead of generating a large number of anchor-based proposals, CenterNet extracts a likelihood map of the image while simultaneously performing bounding box regression. In this framework, keypoints are identified as local likelihood maxima within the heatmap, evaluated using 8-connectivity criteria.

Keypoints and likelihood maps are obtained from different outputs of the encoding and decoding layers. Once keypoints are identified, bounding boxes are directly generated around them. This process eliminates the need for IoU-based NMS, as erroneous or spurious predictions can be effectively discarded through a simple thresholding operation, reducing computational complexity.

The network is trained using a composite loss function, which consists of: A keypoint penalty term, ensuring the model accurately detects object centers. A bounding box size loss, optimizing the dimensions of detected objects. An offset loss, refining the localization of each bounding box’s center. By eliminating anchor boxes and NMS, CenterNet significantly simplifies the object detection pipeline while maintaining high detection accuracy and computational efficiency. This makes it particularly suitable for real-time applications and edge-device deployments.

#### 2.1.6. Benchmarking of Object Detection Networks

In order to provide a reliable and equitable comparison between the different classification architectures, a benchmarking study was performed by [35] on the COCO dataset [36], encompassing 80 categories. This study evaluated the performance of selected architectures on an edge device. The results, in terms on Mean average precision (mAP) are summarized in Table 1:

Although the study provides valuable insight on the accuracy of the different models for multi-class detection problems, it does not evaluate them with regard to their classification speed. Since the overall objective of the present study is to develop a reliable real-time classification system, it was necessary to further analyze the models in terms of classification speed, to evaluate their overall suitability. The results will be presented in Section 4.

### 2.2. Trackers

Multi-object tracking (MOT) is a fundamental task in computer vision, enabling the continuous identification and association of objects across sequential frames in a video stream. While object detection localizes vehicles in each frame, tracking algorithms establish temporal consistency, ensuring that the same vehicle is correctly identified across multiple frames: time consistent identification is fundamental in order to provide robust and reliable vehicle counting data. The integration of a tracker with an object detector must balance precision and computational efficiency, particularly in embedded systems with limited resources. Unlike detection-based approaches that process frames independently, MOT algorithms utilize motion models and association techniques to minimize computational redundancy and improve tracking continuity. However, challenges such as occlusions, motion blur, scale variations, and abrupt trajectory changes can significantly impact tracking performance in complex traffic environments.

Modern tracking algorithms differ based on their association strategy. Traditional methods like SORT (Simple Online and Real-time Tracker, [37]) and DeepSORT [38] use Kalman filters and Hungarian matching, leveraging motion and appearance features to maintain object identity. More recent approaches, including ByteTrack [39] and BOTSort [40], enhance tracking accuracy by incorporating low-confidence detections and motion cues, reducing identity switches and improving performance in dense urban traffic. Given the real-time constraints of edge computing applications, selecting an appropriate tracker involves a trade-off between computational efficiency and tracking robustness, ensuring optimal performance for real-time vehicle monitoring systems.

#### 2.2.1. SORT

Simple Online and Real-time Tracking (SORT) is a lightweight and computationally efficient multi-object tracking (MOT) algorithm designed for real-time applications. Unlike more complex tracking methods that rely on appearance models or deep learning-based feature extraction, SORT adopts a minimalist approach by using only the bounding box position and size for motion estimation and data association. This simplicity enables high-speed tracking performance, making it particularly suitable for embedded systems and resource-constrained environments such as traffic monitoring on edge devices.

SORT operates within a tracking-by-detection framework, where detections from an object detector, such as YOLOv8, are linked across frames to maintain object identity over time. The core tracking mechanism relies on two well-established techniques:

1. A Kalman filter to predict the future positions of objects based on their motion dynamics.
2. The Hungarian algorithm for efficient data association by minimizing the distance between predicted and detected bounding boxes.

One of SORT’s key advantages is its exceptional speed, running at 260 Hz—over 20 times faster than many traditional MOT algorithms. However, this speed comes at the cost of limited robustness to occlusions and identity switches, as it does not incorporate appearance features for object re-identification. In highly crowded or complex traffic scenes, this can lead to frequent ID switches, reducing tracking consistency.

Despite these limitations, SORT remains an optimal choice for real-time vehicle tracking on embedded devices due to its low computational overhead and high processing speed. It is particularly well-suited for applications where speed is prioritized over long-term identity retention, such as vehicle flow counting in urban environments. However, for improved robustness in dense traffic scenarios, enhanced tracking algorithms like DeepSORT or ByteTrack may be more appropriate.

#### 2.2.2. DeepSORT

DeepSORT (Deep Simple Online and Real-time Tracking) extends the original SORT algorithm by incorporating appearance-based re-identification, significantly improving object tracking in occluded and crowded environments. While SORT relies solely on motion-based tracking using Kalman filtering and IoU matching, DeepSORT enhances data association by integrating deep learning-based appearance features, which help maintain object identities even when detections are lost for short periods.

DeepSORT retains the Kalman filter for motion prediction and the Hungarian algorithm for data association but introduces a deep convolutional neural network (CNN) for feature extraction. This CNN is pre-trained on a large-scale re-identification dataset, enabling the tracker to differentiate objects based on their visual appearance rather than just their positional information. This enhancement reduces identity switches by 45% compared to SORT, making it particularly useful for tracking vehicles in traffic monitoring applications, where occlusions frequently occur.

While DeepSORT provides superior tracking accuracy, its reliance on a deep learning model increases computational complexity, making it less suitable for embedded devices with limited resources. The additional CNN-based processing requires GPU acceleration, which may not be feasible for real-time tracking on edge devices such as the Raspberry Pi 4 with a Coral TPU. Thus, while DeepSORT improves tracking robustness, lighter alternatives like ByteTrack or BOTSort may be more appropriate for real-time vehicle tracking applications on embedded systems.

#### 2.2.3. ByteTrack

ByteTrack is a multi-object tracking (MOT) algorithm designed to enhance the accuracy and robustness of tracking-by-detection methods. Unlike conventional trackers that discard low-confidence detections due to thresholding, ByteTrack associates every detection box, improving tracking continuity and reducing identity switches, particularly in cases of occlusion and motion blur. This makes it highly suitable for the monitoring of high volumes of traffic, where vehicle detection performance can be affected by factors such as shadows, overlapping vehicles, and varying illumination conditions.

ByteTrack builds upon SORT by using a two-step association strategy: it first matches high-confidence detections to existing tracklets using IoU-based Hungarian matching, then associates low-confidence detections to recover objects that might have been missed due to occlusion or detection uncertainty. This approach significantly improves MOTA (Multiple Object Tracking Accuracy) and IDF1 (Identity F1 Score), outperforming many state-of-the-art trackers while still allowing for real-time detection.

One of ByteTrack’s key advantages is its computational efficiency, as it relies only on motion-based cues (Kalman filtering) for association, without requiring deep-learning-based re-identification (ReID) models. This makes it suitable for embedded systems and real-time vehicle tracking applications, where processing speed and resource efficiency are critical. Compared to SORT, ByteTrack achieves higher tracking precision while maintaining a comparable processing speed, making it an excellent choice for edge-device deployment in intelligent traffic monitoring systems.

#### 2.2.4. BOTSort

BOTSort (Bag of Tricks SORT) is a multi-object tracking (MOT) algorithm designed to improve upon traditional SORT-based trackers by enhancing motion modeling and data association. Like SORT and ByteTrack, BOTSort follows a tracking-by-detection framework but incorporates additional refinements to reduce ID switches and improve tracking accuracy in complex scenarios, such as crowded urban traffic flows.

BOTSort introduces three key modifications to conventional SORT-based tracking methods:

1. Refined Kalman Filtering—It modifies the state vector of the Kalman filter, leading to more accurate bounding box localization, particularly for vehicles moving at high speeds.
2. Camera Motion Compensation (CMC)—BOTSort accounts for camera-induced motion, ensuring stable tracking even when the camera is moving or vibrating.
3. Improved IoU-Based Association with ReID Fusion—The tracker integrates cosine distance-based re-identification (ReID) with IoU-based matching, creating a more robust association strategy that reduces false reassignments and identity fragmentation.

By integrating these enhancements, BOTSort outperforms ByteTrack and SORT in dense tracking environments, achieving higher MOTA (Multiple Object Tracking Accuracy) and IDF1 scores. Despite these improvements, BOTSort maintains competitive efficiency, making it suitable for real-time traffic monitoring applications on embedded systems.

#### 2.2.5. Benchmarking for Tracking Algorithms

A comparative study of the results presented in literature was conducted in order to select the best possible tracking algorithm in terms of performance. The accuracy of the tracking algorithms was evaluated through the Multiple object tracking accuracy (MOTA) metric [41]. The results of this study are summarized in Table 2:

This study [40] highlighted BOTSort as the most suitable candidate for the development of a robust tracking framework, since it showed the best performance (MOTA = 80.6%) among other popular models.

In light of the analyzed literature and preliminary benchmarks, the positioning of this study emerges from the need to balance high classification accuracy with the strict constraints of low-cost edge computing. While lightweight architectures like SSD MobileNet offer high inference speeds, they often struggle with the fine-grained classification required to distinguish between similar categories (e.g., heavy vehicles vs. buses) as mandated by the CNOSSOS-EU model. Conversely, highly accurate two-stage detectors or heavy models like CenterNet require computational resources unavailable on standard embedded devices. This work addresses this specific gap by selecting the YOLOv8 architecture optimized through post-training quantization integrated with a resource-efficient tracking algorithm. This combination is designed to deliver the precision of high-end commercial systems while maintaining the cost-effectiveness and versatility required for large-scale acoustic monitoring networks.

## 3. Methodology

This section provides a detailed overview of the low cost object detection system’s development. It begins with a description of the experimental setup, outlining each system component and its role. Next, the dataset is analyzed, focusing on its characteristics and impact on the network’s training process. A preliminary evaluation of the proposed detection models follows, leading to the selection of the three top-performing architectures for further study. The fourth section introduces tracking algorithms, discussing their integration with the detection system. Finally, the quantization process is presented, detailing its implementation and impact on model efficiency.

### 3.1. Experimental Setup

The experimental setup was based on the previous model presented in [11]. While the previous system was mostly focused on video recording and cost minimization, the current system was developed with the aim of improving its computational power, while also allowing for real-time vehicle detection; thus providing an accurate, simple, compact and cost-effective device.

**Figure 1:**
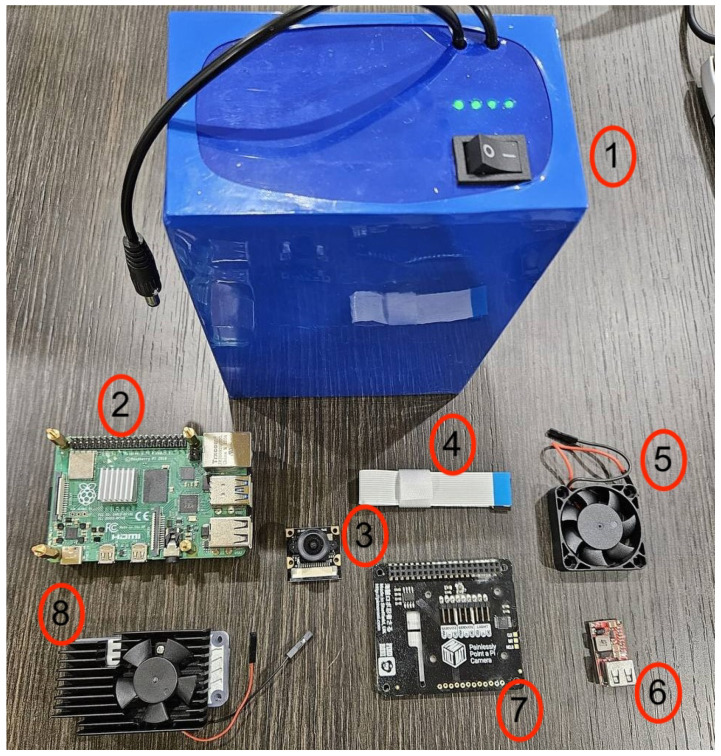
System components: 1. Lithium battery (Laser Electronic Srl, Marano Principato, Italy); 2. Raspberry Pi 4 Model B (Raspberry Pi Ltd., Cambridge, UK); 3. Video camera (Raspberry Pi Ltd., Cambridge, UK); 4. Additional wiring (necessary for Raspberry-camera connection); 5. Cooling fan; 6. Voltage converter (necessary for battery-Raspberry connection); 7. Pan-tilt mechanism (Waveshare Electronics, Shenzhen, China); 8. Coral TPU cooling fan (Google LLC, Mountain View, CA, USA).

In order to obtain the computational speed required for real-time classification, a Raspberry Pi 4 microprocessor with an additional Coral TPU module was utilized. The system was powered by a portable lithium battery connected through a USB-C port, allowing for extended measurement sessions. The Raspberry Pi 4 has an average power consumption of approximately 2.7 W, with values ranging from 1.8 W (idle) to 5.1 W (under full load). The Coral TPU module adds an additional 2 W under maximum load, while its typical consumption remains around 0.5–1 W depending on the processing demand. Considering all components, the system’s total power consumption ranges from 2.3 W (minimum) to 7.1 W (maximum), with an average of approximately 4 W during typical operation. The system’s energy autonomy was estimated to be approximately 72 h for low-energy consumption applications and up to 24 h for high-energy-consumption ones, such as online, real-time classification. A cooling fan was also installed on the device and on the Coral TPU to avoid issues associated with overheating during prolonged measurement sessions. A mechanical device was also installed on the device to allow for camera movement. The entire system was enclosed in a dedicated box to prevent potential atmospheric damage. The different components of the system are visible and briefly summarized in Figure 1.

**Figure 2:**
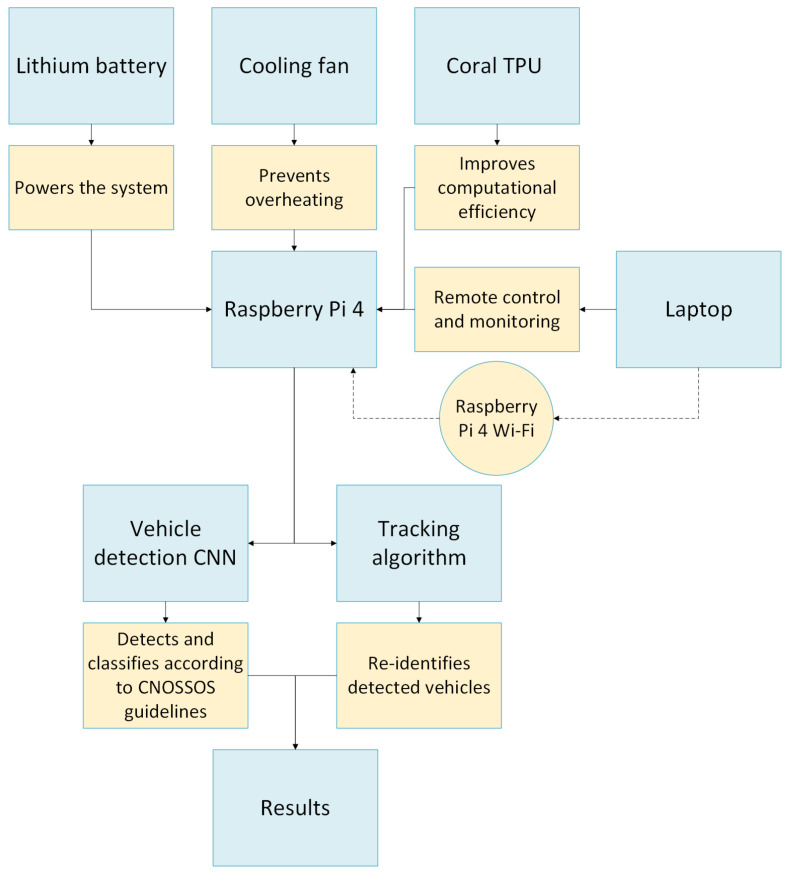
Block diagram providing a synthetic representation of the proposed system architecture and methodology. Solid arrows indicate physical connections and direct data flow between components, while dashed arrows represent the wireless communication via Wi-Fi for remote monitoring and control.

The system can be controlled using a laptop PC without the need for dedicated wiring by leveraging the Wi-Fi capabilities of the Raspberry Pi 4 microprocessor. The proposed methodology is summarized in Figure 2.

### 3.2. Dataset

The training dataset comprised ground-truth-labeled vehicle data, where each detected label was correspondingly associated with the acoustic classes defined by the CNOSSOS-EU model. An additional class, associated with buses, was included, while the open category, included in the CNOSSOS-EU framework, was scrapped. The data set thus contained the following 5 labels:

- Light vehicles: including cars and vans up to 3.5 tons.
- Medium heavy vehicles: including vans with a weight exceeding the 3.5 tons limit.
- Heavy vehicles: Heavy duty vehicles with more than 2 axles.
- Buses: Reserved to heavy buses.
- Powered two wheelers: Motorcycles, tricycles, quads and mopeds.

The custom dataset consisted of 15,000 images (640 × 480 resolution), extracted from video footage collected during measurement campaigns at 18 locations across Italy, including Pisa, Rome, Naples, Piombino, and Cagliari. All data were acquired using our proprietary sensing system, and no external datasets were used. The choice of these cities reflects the logistical feasibility of field deployments and allowed for the inclusion of a representative variety of urban scenarios. Data acquisition was conducted in a controlled and supervised environment, ensuring high quality and variability to improve the generalization capability of the trained models. To capture diverse traffic conditions, images were taken under different lighting conditions: approximately 9000 day time images and 6000 night time images. While vehicles were clearly visible in day time images, night time samples exhibited slight blurring due to sub-optimal lighting conditions.

**Figure 3:**
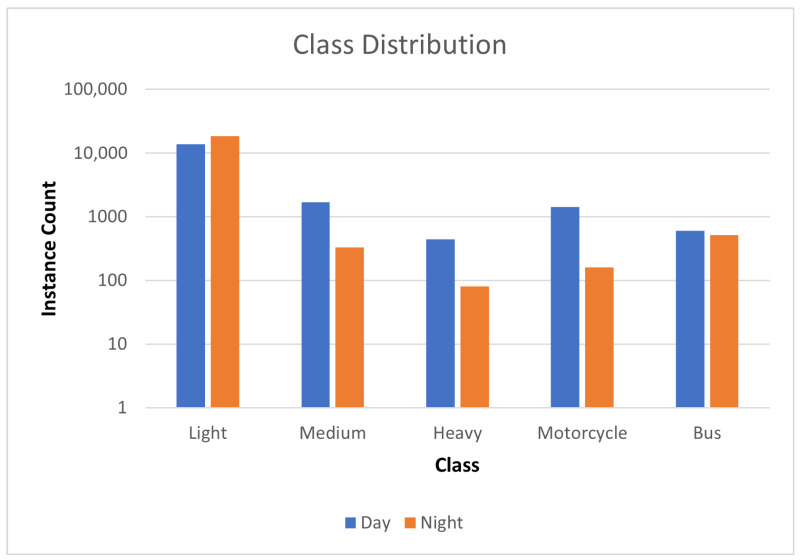
Vehicle class distribution for the refined data set.

The distribution of classes for each vehicle category is illustrated in Figure 3 using a logarithmic scale. It is evident that light vehicles are significantly more prevalent than other categories, reflecting the typical imbalance in traffic circulation. To enhance dataset quality, images with uneven illumination, excessive occlusions, or extreme camera angles were discarded, resulting in a refined dataset of 11,600 images (7056 day time and 5544 night time). The final dataset exhibited a natural imbalance toward the “Light Vehicle” class, reflecting real-world urban traffic distributions. To mitigate this imbalance, Focal Loss was employed, modifying Cross-Entropy Loss to reduce the impact of well-classified examples and improve learning on harder-to-classify instances. The dataset was then split into training (80%), validation (10%), and test sets (10%) for model training and evaluation.

### 3.3. Real-Time Detection and Model Quantization

For this application, the object detection network was deployed on a Raspberry Pi 4, equipped with a Coral TPU accelerator to enable real-time inference. Given the computational constraints of an embedded system, a quantized model was implemented to optimize efficiency while maintaining detection accuracy. Prior research [42] demonstrated that 8-bit integer quantization significantly increases classification speed with minimal accuracy loss, while findings in [43] indicated that 4-bit and 8-bit quantization could improve processing speed by up to eight times while reducing power consumption. Based on these advantages and the Coral TPU’s compatibility with 8-bit models, all networks in this study were subjected to 8-bit post-training quantization.

In order to leverage transfer learning on pre-trained models for custom detection tasks, post-training quantization was preferred over training a fully quantized model from scratch. Since quantization reduces the computational burden by converting model parameters into 8-bit integers, its impact is more pronounced on deeper networks, whereas lighter architectures, such as YOLOv8n, experience minimal degradation. To efficiently quantize the detection model, both network weights and activation values were converted into 8-bit integers.

Quantization can have a significant impact on the computational requirements of the model. CNNs extract information from images through subsequent convolutions with adaptable filters (kernel). The convolutional output at position i,j for a kernel of dimensions M×N con be written as:

(2) $$O\left(i,j\right)=I*K=\sum_{m=0}^{M}\sum_{n=0}^{N}I\left(i+m,j+n\right)K\left(m,n\right)$$

where *O* denotes the output, *I* the starting image and *K* the kernel.

The network is trained by optimizing the trainable parameters of the kernels. The 32-bit floating point number format is usually used to store kernel weights. Quantization reduces the computational burden of the network by converting the kernel weights into 8-bit integers:

(3) $$Q\left(\omega \right)=Int\left(\frac{\omega }{S}-Z\right)$$

where ω stands for the initial weight, Q(ω) for the quantized one, *S* for a scaling factor and *Z* for a bias factor. Both *Z* and *S* are determined by the maximum and minimum values of the weights and the number of bits used for quantization.

To ensure a precise and reliable evaluation of the traffic flow for each class, the CNN was coupled with a robust multi-object tracking (MOT) algorithm. This association allows the system to assign a unique and persistent ID to each detection, preventing multiple counts for the same vehicle. Among the tracking algorithms discussed in Section 2, BoT-SORT was selected for the final implementation. This choice was driven by the need to balance computational efficiency on edge devices with tracking robustness in complex urban scenarios. Specifically, BoT-SORT’s integration of camera motion compensation and its ability to recover tracks from low-score detection boxes proved essential in maintaining consistent vehicle IDs during partial occlusions or when vehicles were moving away from the camera’s optimal focal point. This setup ensures real-time processing, as the tracking logic, although based on Kalman filters and global data association, was optimized to run alongside the hardware-accelerated inference

Kalman filters are a two-step (prediction and update) method for the description and prediction of moving objects. Kalman filters aim at providing reliable predictions by minimizing the associated mean squared error.

Given a state vector (usually a seven-tuple or an eight tuple containing positions, bounding box dimensions and their time derivatives) for each object of interest, it is possible to predict the possible future state and its associated covariance:

(4) $${\hat{x}}_{k|k-1}^{p}=M_{k-1}^{k}{\hat{x}}_{k|k-1}^{o}+T_{k-1}^{k}$$

where $\hat{x^{p}}$ stands for the predicted state vector; $\hat{x^{o}}$ for the observed state vector; *M* for the state transformation matrix and *T* for the translation transformation vector.

(5) $$P_{k|k-1}^{p}=M_{k-1}^{k}P_{k|k-1}^{o}{\left(M_{k-1}^{k}\right)}^{T}$$

where $P^{p}$ stands for the predicted covariance matrix and $P^{o}$ for the observed one.

Observations are then compared with the predicted value in order to update and optimize the Kalman filter. Given an observation matrix $H_{k}$ that transforms the observed state vector (seven-tuple or eight-tuple) into the observation vector *z* (four tuple) and the measurement noise covariance $R_{k}$, it is possible to describe the update process of a Kalman filter as:

(6) $$K_{k}=P_{k|k-1}^{P}H_{k}^{T}{\left(H_{k}P_{k|k-1}^{p}H_{k}^{T}+R_{k}\right)}^{-1}$$

(7) $${\hat{x}}_{k|k}^{o}={\hat{x}}_{k|k-1}^{p}+K_{k}\left(z_{k}-H_{k}{\hat{x}}_{k|k-1}^{p}\right)$$

(8) $$P_{k|k}^{o}=\left(I-K_{k}H_{k}\right)P_{k|k-1}^{p}$$

*K* is called Kalman gain.

Additionally, input calibration data was required to estimate the range of floating-point tensors, including model inputs, activations, and outputs. This calibration was conducted using 300 reference images, ensuring that the final models remained fully compatible with the Coral TPU accelerator.

Vehicle counting is performed using a region-of-interest (ROI) entry approach. A vehicle is counted once when the centroid of its tracked bounding box enters a predefined ROI placed within the camera field of view. Each tracked object is associated with a unique track ID, and a binary flag is used to ensure that each vehicle contributes only once to the final count, even in the presence of stop-and-go traffic conditions or partial occlusions. The ROI is selected to minimize perspective distortions and to ensure that only vehicles fully visible within the scene are counted. Manual counting performed by an operator on the same video segments is used as ground truth for validation.

## 4. Results and Discussion

In the following section, after a preliminary analysis aimed at finding the most suitable models, the results and discussion focus on model selection in terms of mean Average Precision (mAP) across various lighting conditions, as well as a comparison between full and quantized models. Additionally, a real-world case study is presented, wherein the model’s performance is evaluated against ground truth data obtained through manual video annotation, observations from an on-site human operator, and a commercial traffic flow estimation system employed by the municipality. The commercial traffic monitoring system used for comparison is a laser-based counter already installed at the measurement site and routinely employed by the local municipality. The system estimates vehicle categories primarily from the measured vehicle length, a widely adopted approach in infrastructure-grade traffic monitoring solutions. In this study, the performance of the commercial system is evaluated using the absolute error between the aggregated vehicle counts provided by the device and the corresponding ground-truth manual counts. However, length-based classification intrinsically limits the achievable level of detail, as vehicle length does not uniquely map to the CNOSSOS-EU categories. In particular, the discrimination between medium and heavy vehicles, which is critical for acoustic noise modeling, cannot be reliably achieved from length information alone due to overlapping size ranges among different vehicle types. Consequently, while the commercial system is effective for generic traffic flow estimation, it is not designed to provide CNOSSOS-EU–compliant vehicle classification, and its results are therefore included as a representative baseline rather than as a noise-oriented reference solution.

In real life applications, occlusion may represent a serious challenge, as underlined by studies by [44,45]: in order to reduce its effects, the positioning of the measurement camera was carefully considered. In particular, a minimum mounting height of 6 m was employed, as suggested by [45]. Future studies will specifically address this problem, with the aim of finding an optimal and satisfactory solution.

### 4.1. Preliminary Analysis

In order to further assess the overall suitability of different classification models for the development of a real time vehicle identification system, it was necessary to perform a test to evaluate the FPS of the different networks. A global benchmarking process was performed, building up on the foundation illustrated in Section 2.1.6. This further testing allowed for a thorough characterization of the networks, thus providing information on accuracy and classification speed. Table 3 provides a synthetic comparative analysis of all the models evaluated during the initial development phase. In the proposed implementation, retraining the model does not affect inference latency, as the same network architecture, input resolution, and quantized inference pipeline are preserved [46].

From the table the following can be inferred:

- SSDMobileNet: The lightest model tested, evaluated with image sizes of 320 px and 640 px. The standard version, without the Feature Pyramid Network (FPN), achieved 27 FPS with 320 px images and 17 FPS with 640 px images. The inclusion of FPN reduced speed to 18 FPS but improved accuracy, particularly in detecting vehicles. These preliminary tests provided valuable insights for selecting promising models even without specific training for the use case.
- EfficientDet: Although more accurate than SSDMobileNet, this model exhibited significantly lower performance, with speeds of approximately 6 FPS for 320 px images. This limited speed renders it unsuitable for real-time detection but potentially viable for offline applications.
- CenterNet: Tested with ResNet50 and 520 px images, this model faced significant compatibility challenges with the Coral Edge TPU. The quantization process was suboptimal, necessitating execution on the CPU, which resulted in extremely low speeds (around 1 FPS), making it impractical for the intended application.
- YOLO: Evaluated across multiple variants (8n, 8s, 8m, 8l, 8x). The analysis focused on finding the best trade-off between input image size, model complexity, and FPS. The 8n variant, with 448 px images, achieved the best balance at 14 FPS. However, other variants demonstrated lower performance (e.g., 7 FPS for 8s), and the more complex variants saturated the TPU, making them inapplicable.
- RetinaNet: Despite offering good accuracy, this model was unsuitable due to its limited speed of approximately 4 FPS. Considering the primary goal of vehicle counting on a road segment, such low speed could result in missed detections of fast-moving vehicles. Consequently, models capable of processing at least 10 FPS were deemed necessary.

The analysis indicates that models such as SSDMobileNet, SSDMobileNet FPN, and YOLO8n achieve a promising balance between speed, size, and accuracy, rendering them well-suited for the task at hand.

### 4.2. Models Performance

As discussed in the preceding paragraphs, the models selected for training with the dataset include YOLO, SSD MobileNet, and SSD MobileNetFPN. The initial results aim to compare the performance of these three models, as well as their respective quantized versions.

#### 4.2.1. Day Time

Table 4 and Table 5 present the achieved results in terms of mAP_50_ and mAP_50-95_ under day time conditions. The results are further categorized based on the different classes of detected objects, including light, medium, heavy, motorcycle, and bus, with an additional “all” category representing the overall performance across all object types.

The two tables provide a comparative evaluation of YOLO, SSD MobileNet, and SSD MobileNetFPN models under day time conditions, assessed using mAP_50_ and mAP_50-95_. mAP_50_ measures object detection accuracy at an IoU threshold of 50%, making it a more tolerant metric that primarily evaluates detection presence. mAP_50-95_ averages accuracy across multiple IoU thresholds (0.50 to 0.95), making it stricter and more indicative of a model’s localization precision.

From the Table 4 and Table 5 it can appreciated how YOLO achieves the highest mAP_50_ (96.6%) and mAP_50-95_ (86.4%), outperforming SSD-based models across all object categories. SSD MobileNetFPN performs better than SSD MobileNet but remains behind YOLO, particularly in terms of mAP_50-95_, suggesting YOLO’s superior localization precision. There is a significant drop between mAP_50_ and mAP_50-95_ across all models, showing that accurate localization remains challenging, even under good lighting conditions.

Light and medium vehicles have the highest accuracy across all models, indicating that these objects are generally easier to detect. Motorcycle detection remains a challenge, with YOLO dropping from mAP_50_ (83.9%) to mAP_50-95_ (57.8%), suggesting difficulties in achieving precise bounding boxes for small and intricate objects. Heavy vehicles and buses maintain relatively high accuracy, with YOLO performing best, showing its strength in detecting large objects.

Quantization leads to an expected decline in performance across all models, with SSD MobileNet being the most affected. YOLO maintains the highest accuracy post-quantization mAP_50_ (92.2%) and mAP_50-95_ (71.0%), demonstrating better resilience to the precision reduction. SSD MobileNetFPN performs better than SSD MobileNet, confirming that its additional feature pyramid structure helps retain more information post-quantization.

Light vehicles remain the most accurately detected category across all models, as they are the most common and structurally well-defined objects in the dataset. Motorcycles and heavy vehicles experience significant performance degradation, with SSD MobileNet dropping to mAP_50-95_ (48.0%) for motorcycles, further decreasing its accuracy for small objects. YOLO remains the best across all classes post-quantization, showing its superior ability to adapt to reduced numerical precision.

**Figure 4:**
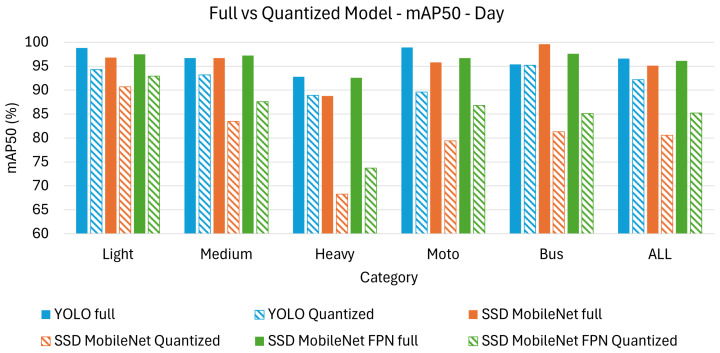
Bar chart comparison for mAP_50_ on day test set.

**Figure 5:**
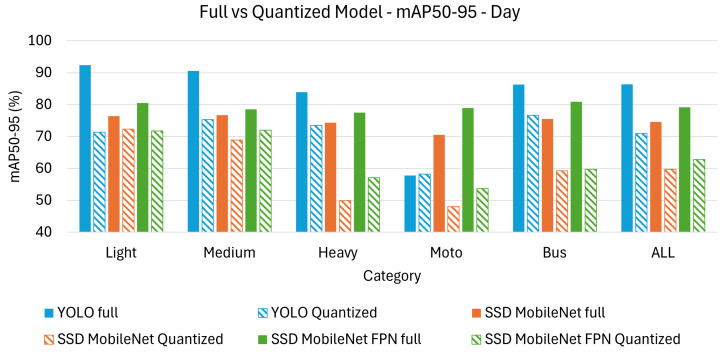
Bar chart comparison for mAP_50-95_ on day test set.

Figure 4 displays a bar chart comparison, visually highlighting the results from Table 4, while Figure 5 does the same for the results from Table 5 using the mAP_50-95_ metric. It is clear that the performance gap between full and quantized models varies across architectures, with YOLO demonstrating greater robustness to quantization compared to SSD MobileNet and SSD MobileNetFPN when analyzing the mAP_50_.

#### 4.2.2. Night Time

On the other hand, Table 6 and Table 7 present the achieved results in terms of mAP_50_ and mAP_50-95_ under night time conditions.

Since night time conditions introduce additional challenges, such as reduced visibility, lower contrast, and increased noise, the analysis will focus on how these factors impact model performance. Where applicable, insights from the day time analysis remain valid and can be referenced for comparison without repetition.

Among the full models, YOLO remains the best-performing option, achieving the highest accuracy across both metrics. The overall performance of all models is lower at night compared to day time conditions, showing that lower illumination significantly impacts object detection. SSD MobileNet suffers the most from night time conditions, dropping to mAP_50_ (74.4%) and mAP_50-95_ (59.3%), highlighting its limitations in extracting meaningful features in low-light scenarios.

Regarding class observations, heavy vehicles continue to be the most accurately detected objects across all models, likely due to their large size, distinct shape, and strong headlights, which provide better contrast. Motorcycles remain the most challenging category, with YOLO scoring mAP_50_ (92.3%) but only mAP_50-95_ (64.5%), reinforcing the difficulty of detecting smaller objects in dark environments. Medium vehicles see a sharper performance decline compared to day time, particularly for SSD MobileNet, going from mAP_50-95_ (45.4%) to mAP_50-95_ (30.7%), suggesting that vehicles with less distinct lighting features are harder to recognize in low light.

For the quantized models, the difficulties of night time object detection are further exacerbated, with SSD MobileNet experiencing the most severe performance drop. YOLO maintains the highest accuracy post-quantization, but the gap between mAP_50_ and mAP_50-95_ widens, indicating that high-IoU object localization is more challenging at night. Motorcycles and medium vehicles remain the most affected categories, with SSD MobileNet reaching only mAP_50-95_ (27.6%) for motorcycles and mAP_50-95_ (18.2%) for medium vehicles, reinforcing the earlier observation that feature extraction struggles under low-light and lower-precision settings.

**Figure 6:**
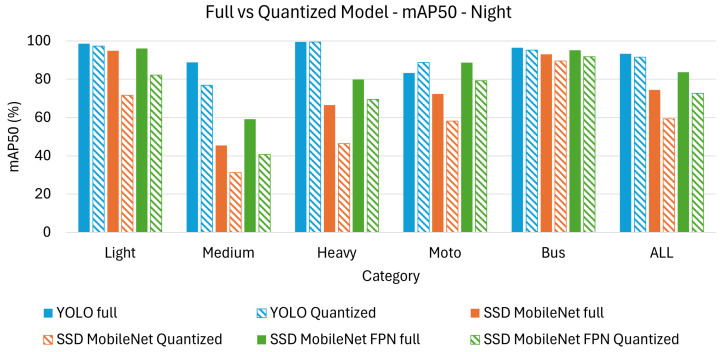
Bar chart comparison for mAP_50_ on night test set.

**Figure 7:**
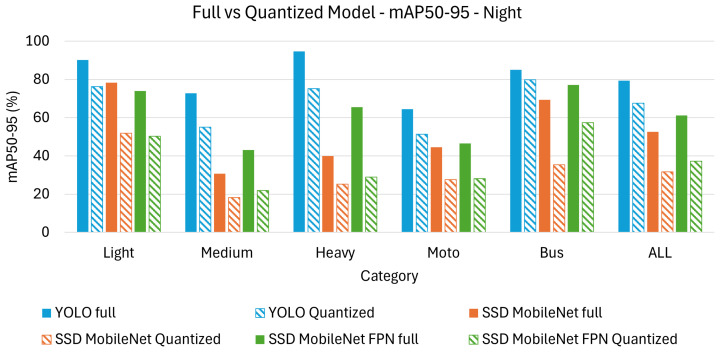
Bar chart comparison for mAP_50-95_ on night test set.

Finally, Figure 6 displays a bar chart comparison, visually highlighting the results from Table 6, while Figure 7 does the same for the results from Table 7. It is clear that the performance gap between full and quantized models varies across architectures, with YOLO demonstrating greater robustness to quantization compared to SSD MobileNet and SSD MobileNetFPN.

Regarding class observations in quantized models, light vehicles perform better compared to other categories, since they generally have illuminated headlights and a well-defined shape, making them easier to detect. Heavy and bus categories maintain relatively higher accuracy than medium and motorcycle classes, showing that larger vehicles are less affected by night time conditions and quantization.

The impact of low-light conditions is significant across all models, but YOLO remains the most robust option. High IoU thresholds (mAP_50-95_) are particularly difficult at night, furhter complicating the challenge of precise object localization. Quantization has a stronger negative effect on night time detection than in day time, particularly for SSD MobileNet, which struggles to retain object features under both low light and reduced numerical precision. YOLO remains the best choice for night time detection, as it maintains the highest accuracy across different IoU thresholds and quantized versions.

### 4.3. System Deployment and Case Study

In order to test the system in a real-world scenario an ad-hoc measurement campaign was planned. The study consisted in setting-up the system in an urban space where a mix of the desired traffic flow (i.e.: light, medium, heavy, bus, motorcycles) was present in both directions. Moreover, in order to validate the results, extra information regarding the traffic vehicle count was needed, therefore human operators manually marked each passing vehicle for each category for six hours. Additionally, a manual frame-by-frame count was performed a posteriori re-watching the video footage to establish the ground truth data. Lastly, an existing commercial traffic monitoring system, used by the municipality present in the area was also used.

**Figure 8:**
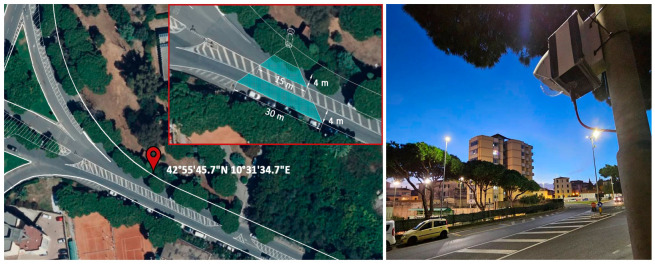
Real-world scenario position and system overview.

Figure 8 presents the real scenario for the city of Piombino. The coordinates of the measurement point are 42°55′45.7″ N 10°31′34.7″ E (WGS84).

The primary objective of this experimental test was to compare the performance of the selected model in both versions: YOLO_full_ and YOLO_Quantizied_. Video recordings were captured during the test for this purpose. The quantized model was implemented in real-time using a Raspberry Pi 4 in conjunction with a Coral accelerator, while the full model was executed using an NVIDIA T4 GPU.

**Figure 9:**
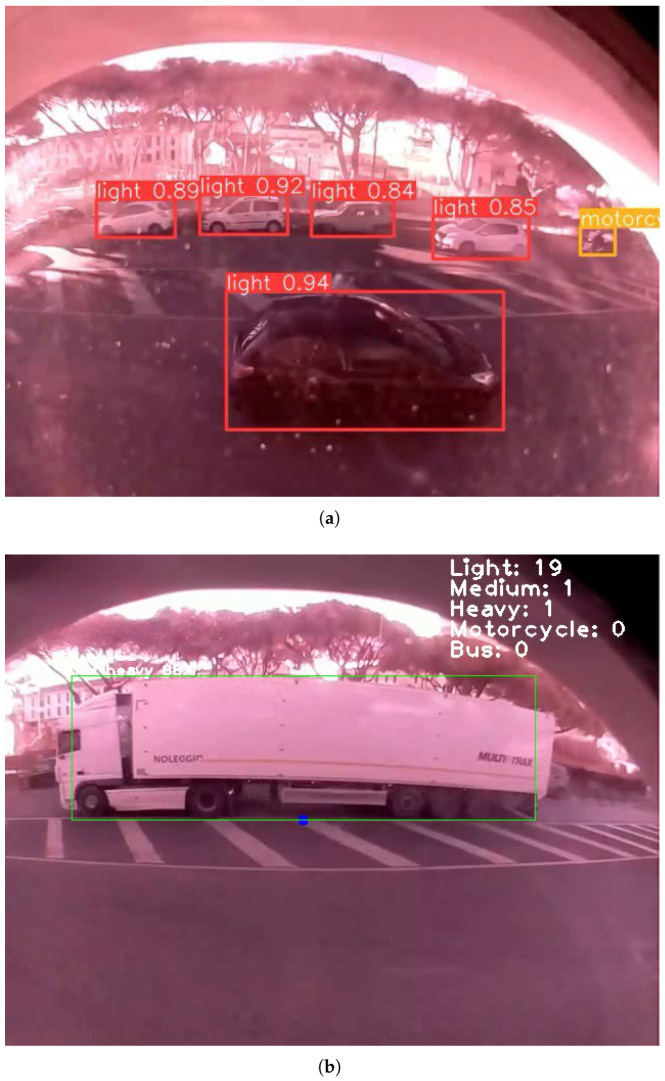
Vehicle detection results obtained using the quantized model in a daytime scenario. (**a**) Detection of light vehicles and motorcycles using the quantized model in a daytime scenario. (**b**) Detection of heavy vehicles using the quantized model in a daytime scenario.

Figure 9 illustrates two frames from the video footage, displaying the associated bounding boxes, predicted category class, and corresponding probability score assigned by the algorithm. As observed in the figures, the algorithm effectively detects vehicles even under challenging conditions. This is particularly evident in Figure 9, where a motorcycle is barely visible on the right side of the frame, sunlight interference is present, and dust from prior weather events partially obstructs the camera dome.

The results of the campaign measurement are summarized in Table 8, which presents a comparative analysis of vehicle counts recorded over a six-hour period. The evaluation encompasses a variety of realistic traffic conditions, ranging from light to dense traffic, including the presence of queues. The table includes data from the Ground Truth, human operators counting, the commercial system currently employed by the municipality of Piombino, the full YOLO model in post-processing and the quantized version of it. The vehicle counts are categorized according to the CNOSSOS-EU classification, distinguishing between heavy vehicles and buses. Additionally, for each category, the total number of detected vehicles and the absolute error—calculated as the sum of absolute deviations in class counts compared to the ground truth were documented.

As can be inferred:

- Human Operators: The total vehicle count recorded by human operators (1799) closely aligns with the Ground Truth (1794), with only a minor overall absolute error of 25. Deviations per category were minimal, with slight under counts for light vehicles (−10) and slight over counts for other classes, including motorcycles (+6) and medium vehicles (+9).
- Commercial System: The commercial system demonstrated significant discrepancies, with a notably lower total vehicle count (1378) compared to the Ground Truth. This was primarily due to large underestimations for light vehicles (−676) and motorcycles (−66). However, the system overestimated heavy vehicles (+287) and medium vehicles (+42), resulting in the highest absolute error (1074).
- YOLO_full_ Model: The YOLO_full_ model exhibited a strong performance, with a total vehicle count of 1869 and an overall absolute error of 75. It closely matched the Ground Truth in most categories, particularly in detecting motorcycles (+6) and buses (0 error). The primary discrepancies were a slight overestimation in heavy vehicles (+27) and medium vehicles (+31).
- YOLO_quantized_ Model: The YOLO_quantized_ model exhibited slightly higher deviations, with a total vehicle count of 1723 and an absolute error of 119. This model tended to underestimate light vehicles (−53) and motorcycles (−36) while overestimating medium vehicles (+24).

The commercial system used as baseline in this study is an on-site laser-based traffic counter already installed at the measurement location. The device assigns vehicle classes primarily from the measured vehicle length, which is a widely adopted proxy in operational deployments. In this work, the performance of the commercial baseline is expressed through the absolute error between the aggregated counts returned by the device and the corresponding reference counts. Nevertheless, a length-only approach inherently limits the achievable classification granularity. Vehicle length does not uniquely map to the CNOSSOS-EU categories because different vehicle types may share overlapping length ranges. Most importantly, the separation between medium and heavy vehicles, which is critical for CNOSSOS-EU compliant outputs, cannot be robustly inferred from length alone. This limitation becomes particularly relevant when the target is the full set of five CNOSSOS-EU vehicle categories. For completeness, it is worth noting that, even among video-based traffic recognition solutions, the adopted taxonomies are typically application-driven (e.g., car, van, truck, bus, motorcycle) rather than CNOSSOS-EU oriented, and therefore they seldom address the medium versus heavy ambiguity required by noise modelling frameworks. Table 9 presents the weighted percentage absolute error for each detection method. It is important to consider that the statistical distribution of the data aligns with the typical distribution found in urban environments. Consequently, the influence on traffic estimation must be proportional to the number of instances present in the statistical sample. To achieve this, the weights correspond to the proportion of each vehicle class relative to the total traffic flow, ensuring that errors in more frequent vehicle categories (e.g., light vehicles) have a greater impact on the overall error percentage. Therefore Table 9 provides weighted columns that normalize the importance of each class, providing a clearer understanding of the relative significance of different object categories rather than just raw counts.

Results presented in the table can thus be summarized:

- Human Operators: The weighted percentage absolute error is minimal (1.4%), highlighting the accuracy of manual counting.
- Commercial System: The error is disproportionately high (59.9%), mainly due to extreme deviations in light (37.7%) and heavy (16.0%) vehicle counts.
- YOLO_full_ Model: This model maintains a general low error rate (4.2%), performing particularly well in detecting light vehicles and motorcycles.
- YOLO_quantized_ Model: The quantized version has a slightly higher overall error (6.6%) but remains significantly more accurate than the commercial system.

As expected, the human operator’s count closely aligns with the Ground Truth, while the YOLO_full_ model exhibited a slightly higher absolute error. In contrast, the commercial system demonstrated significant inaccuracies, notably overestimating the count of heavy vehicles by nearly six times the actual number and underestimating light vehicles, detecting only about half of their true count. The quantized model, in this case, showed a tendency to underestimate light vehicles and motorcycles. Its overall error was higher than that of the full model, likely due to reduced detection accuracy caused by quantization. Despite this decline in performance, the quantized model still outperformed the commercial system by a significant margin.

The main limitation concerns the categorization of medium-sized and smaller vehicles, especially motorcycles. During testing, cars and vans were sometimes confused with light or heavy categories because of structural similarities, indicating that further model refinement and additional representative data are needed to improve class separation. The system was not evaluated under adverse weather conditions (e.g., fog or heavy rain), as these were not encountered during data collection. Moreover, reliable operation in low-light environments remains critical for real-world deployments. Finally, hardware miniaturization, while beneficial in terms of cost and power consumption, currently limits the use of more sophisticated models and the integration of additional sensing technologies (e.g., radar or lidar).

## 5. Conclusions

A real-time multi-vehicle recognition system was developed by combining low-cost embedded hardware (Raspberry Pi 4 plus Coral TPU) with an optimized deep-learning pipeline for traffic categorization consistent with CNOSSOS-EU. The tests confirmed real-time operation without video post-processing. After 8-bit post-training quantization, the detector runs at 14 FPS and reaches mAP@50 of 92.2% in daytime conditions. In the real-world case study, the proposed system achieved a weighted percentage error of 6.6%, compared to 59.9% for the commercial system, approaching manual counting (1.4% error). These results validate the effectiveness of the model as a practical tool for traffic flow characterization in support of urban road-noise assessment. A key contribution is the reduction of energy demand and computational load enabled by quantization, which makes the platform affordable, low-power, and scalable for resource-constrained deployments. The system also showed robust recognition of large vehicles (buses and trucks).

Future research will focus on addressing these limitations. The integration of infrared cameras represents a promising direction to enhance detection performance under constrained visibility conditions. In addition, the adoption of higher-performance embedded components will be considered to enable more advanced models and multi-sensor configurations. A further development will involve estimating the mean vehicle speed for each category, thereby improving the usefulness of the outputs for decision-makers and enhancing the accuracy of refined CNOSSOS-EU inputs. This extension will require careful camera calibration and robust computer vision strategies for speed estimation, as errors in detection or tracking directly propagate to speed measurements.

In conclusion, the proposed solution demonstrates the feasibility of low-cost, edge-based traffic monitoring systems for practical traffic characterization tasks, supporting acoustic modelling applications without relying on expensive or invasive sensing infrastructures. 

## Figures and Tables

**Table 1:** Performance comparison of different object detection models in terms of mAP on the COCO dataset (80 classes).

Network	mAP (COCO Dataset)
SSD MobileNet	20.2
EfficientDet	33.6
CenterNet	29.5
YOLOv8	37.3
RetinaNet	34.3

**Table 2:** Performance comparison of tracking algorithms based on Multiple Object Tracking Accuracy (MOTA, IDF1).

Tracker	MOTA (%)	IDF1
SORT	74.6	76.9
DeepSORT	75.4	77.2
ByteTrack	80.2	77.3
BOTSort	80.6	79.5

**Table 3:** Performance comparison for different object detection models.

Models	mAP	FPS	Inference Time (ms)	Input Size (px)
SSDMobileNet	20.2	27	37.03	320
SSDMobileDet	23.5	20	50.00	320
SSDMobileNet FPN	29.1	18	55.55	640
YOLO	37.3	14	71.42	448
EfficientDet	33.6	6	166.66	320
RetinaNet	34.3	4	250.00	640
CenterNet	29.5	1	833.33	520

**Table 4:** Models’ mAP_50_ comparison on the day test set.

	Full Model			Quantized		
	**YOLO**	**SSD** **MobileNet**	**SSD** **MobileNetFPN**	**YOLO**	**SSD** **MobileNet**	**SSD** **MobileNetFPN**
Light	98.8	96.8	97.5	94.3	90.7	92.9
Medium	96.7	96.7	97.2	93.2	83.5	87.6
Heavy	92.8	88.8	92.6	88.9	68.3	73.7
Motorcycle	98.9	95.8	96.7	89.6	79.4	86.8
Bus	95.4	99.6	97.6	95.2	81.3	85.1
All	96.6	95.1	96.1	92.2	80.6	85.2

**Table 5:** Models’ mAP_50-95_ comparison on the day test set.

	Full Model			Quantized		
	**YOLO**	**SSD** **MobileNet**	**SSD** **MobileNetFPN**	**YOLO**	**SSD** **MobileNet**	**SSD** **MobileNetFPN**
Light	92.4	76.4	80.5	71.4	72.3	71.8
Medium	90.6	76.7	78.5	75.3	68.9	72.0
Heavy	83.9	74.3	77.5	73.5	49.9	57.1
Motorcycle	57.8	70.5	78.9	58.2	48.0	53.7
Bus	86.3	75.5	80.9	76.7	59.3	59.7
All	86.4	74.6	79.2	71.0	59.7	62.8

**Table 6:** Models’ mAP_50_ comparison on the night test set.

	Full Model			Quantized		
	**YOLO**	**SSD** **MobileNet**	**SSD** **MobileNetFPN**	**YOLO**	**SSD** **MobileNet**	**SSD** **MobileNetFPN**
Light	98.6	94.9	96.1	97.3	71.6	82.2
Medium	88.8	45.4	59.1	76.9	31.2	40.8
Heavy	99.5	66.6	79.8	99.5	46.4	69.5
Motorcycle	83.3	72.4	88.7	88.8	58.1	79.3
Bus	96.5	93.1	95.2	95.2	89.5	91.8
all	93.3	74.4	83.7	91.5	59.3	72.7

**Table 7:** Models’ mAP_50-95_ comparison on the night test set.

	Full Model			Quantized		
	**YOLO**	**SSD** **MobileNet**	**SSD** **MobileNetFPN**	**YOLO**	**SSD** **MobileNet**	**SSD** **MobileNetFPN**
Light	90.2	78.4	74.0	76.3	51.9	50.3
Medium	72.8	30.7	43.1	55.1	18.2	21.9
Heavy	84.7	40.0	65.5	75.2	25.3	28.9
Motorcycle	64.5	44.6	46.6	51.4	27.6	28.1
Bus	85.1	69.3	77.1	79.9	35.4	57.4
All	79.4	52.6	61.2	67.6	31.7	37.3

**Table 8:** Comparison of object counts across different detection systems. (Differences in brackets refer to ground truth.)

Class	Ground	Human	Commercial	YOLO	YOLO
	Truth	Operator	System	Full	Quantized
Light	1574	1564 (−10)	898 (−676)	1585 (+11)	1521 (−53)
Medium	62	71 (+9)	104 (+42)	93 (+31)	86 (+24)
Heavy	44	44 (0)	331 (+287)	71 (+27)	38 (−6)
Motorcycle	111	117 (+6)	45 (−66)	117 (+6)	75 (−36)
Bus	3	3 (0)	-	3 (0)	3 (0)
All	1794	1799	1378	1869	1723
Abs error	0	25	1074	75	119

**Table 9:** Real-word scenario results comparison in terms of weighted percentage error.

Class	Ground	Human	Commercial	YOLO	YOLO
	Truth	Operator	System	Full	Quantized
Light	0.877	0.6%	37.7%	0.6%	3.0%
Medium	0.035	0.5%	2.3%	1.7%	1.3%
Heavy	0.025	0.0%	16.0%	1.5%	0.3%
Motorcycle	0.062	0.3%	3.7%	0.3%	2.0%
Bus	0.002	0.0%	0.2%	0.0%	0.0%
All	1.000	1.4%	59.9%	4.2%	6.6%

## Data Availability

The original contributions presented in this study are included in the article. Further inquiries can be directed to the corresponding author.
